# Locating Youth Exposed to Parental Justice Involvement in the Electronic Health Record: Development of a Natural Language Processing Model

**DOI:** 10.2196/33614

**Published:** 2022-03-21

**Authors:** Samantha Boch, Syed-Amad Hussain, Sven Bambach, Cameron DeShetler, Deena Chisolm, Simon Linwood

**Affiliations:** 1 College of Nursing University of Cincinnati Cincinnati, OH United States; 2 James M Anderson Center for Health Systems Excellence Cincinnati Children's Hospital Medical Center Cincinnati, OH United States; 3 IT Research and Innovation Abigail Wexner Research Institute Nationwide Children's Hospital Columbus, OH United States; 4 Biomedical Engineering Undergraduate Department Notre Dame University Notre Dame, IN United States; 5 College of Medicine and Public Health, College of Nursing The Ohio State University Columbus, OH United States; 6 Nationwide Children's Hospital Columbus, OH United States; 7 School of Medicine University of California Riverside, CA United States

**Keywords:** parental incarceration, machine learning, natural language processing, parental justice involvement, adverse childhood experiences, pediatrics, pediatric health, parenting, digital health, electronic health record, eHealth

## Abstract

**Background:**

Parental justice involvement (eg, prison, jail, parole, or probation) is an unfortunately common and disruptive household adversity for many US youths, disproportionately affecting families of color and rural families. Data on this adversity has not been captured routinely in pediatric health care settings, and if it is, it is not discrete nor able to be readily analyzed for purposes of research.

**Objective:**

In this study, we outline our process training a state-of-the-art natural language processing model using unstructured clinician notes of one large pediatric health system to identify patients who have experienced a justice-involved parent.

**Methods:**

Using the electronic health record database of a large Midwestern pediatric hospital-based institution from 2011-2019, we located clinician notes (of any type and written by any type of provider) that were likely to contain such evidence of family justice involvement via a justice-keyword search (eg, prison and jail). To train and validate the model, we used a labeled data set of 7500 clinician notes identifying whether the patient was ever exposed to parental justice involvement. We calculated the precision and recall of the model and compared those rates to the keyword search.

**Results:**

The development of the machine learning model increased the precision (positive predictive value) of locating children affected by parental justice involvement in the electronic health record from 61% (a simple keyword search) to 92%.

**Conclusions:**

The use of machine learning may be a feasible approach to addressing the gaps in our understanding of the health and health services of underrepresented youth who encounter childhood adversities not routinely captured—particularly for children of justice-involved parents.

## Introduction

Parental justice involvement (eg, prison, jail, parole, or probation) is an unfortunately common and disruptive household adversity for many youths in the United States. Over 5.7 million US children, or nearly 1 in every 14 youth, have experienced a parent’s incarceration in jail or prison, and these are disproportionately youth of color, youth in poverty, and youth in rural areas [1]. Even worse, nearly half of all US children have at least one parent with a record of crime which can affect where a family lives, works, and their eligibility for governmental economic assistance [2]. Children of incarcerated parents are at risk for out-of-home placement [3,4], delinquency [5], poor behavioral health symptoms, [5-7], and school problems [8] with challenges lasting through adulthood [9,10]. The National Academies of Sciences, Engineering, and Medicine (NASEM) [11,12] and the US Department of Health and Human Services [13] have recently advocated for greater information on these youths to inform when, how, and where to best support their health and well-being [11,13]. However, data on this adversity is not routinely collected in pediatric health care settings (and if it is, it is not discrete and unable to be readily analyzed), so we know very little about these youths using reliable measures of health. Because of these gaps [7], few efforts exist to track or facilitate timely follow-up with the remaining children when a parent is arrested (or incarcerated) in order to link comprehensive family support services that could likely mitigate these poor outcomes. Until routine screening on this adversity or novel, cross-sectorial data linkages with justice/court systems become commonplace, leveraging data science tools are feasible, timely, and cost-effective.

Due to advances in artificial intelligence, researchers are learning to leverage clinician notes and other text in the electronic health record to assist in identifying families affected by the social determinants of health. Prior work using natural language processing and machine learning to extract social risk information from clinical notes of adult patients in the United States has been effective [14-17]; however, there is limited use of this work within pediatric settings. The use of machine learning-based algorithms in pediatric medicine has been explored to optimize detection/diagnosis, treatment, and outcome/risk predictions in children who suffer from specific conditions such as severe sepsis [18], autism spectrum disorder [19-22], traumatic brain injuries [23], substance use disorder [24], and asthma [25]. The benefits and drawbacks of their usage in pediatric clinical care have been described by others [26,27]. The application of these techniques to advance our understanding of the health and clinical care of youth who suffer from various adversities holds great promise, yet, few have leveraged such approaches in pediatric research.

To our knowledge, only one study explored the use of natural language processing to locate adults with a history of personal incarceration using the Veterans Administration health record [28]. No study, to date, has examined the use of natural language processing to locate children of justice-involved parents, absent self-report screening tools, nor has research leveraged advanced machine learning models to enhance model accuracy. A recently published study (led by the first author) appears to be the first to leverage natural language processing tools to identify children with any type of contact with the justice system (personal or family) in one large pediatric system [29]. Despite these youths making up only 2% of the pediatric population, they accounted for more than half of substance use and trauma-related diagnoses, nearly half of all stress-related diagnoses, and a third of all psychiatric disorders and suicide-related diagnoses within this institution spanning a 14 year time period [29]. A closer review of 1000 random clinician notes pulled from the search revealed that the exposure to parental incarceration was the most frequent type of justice involvement [29]. These findings, in combination with the identified gaps in the sciences on children of justice-involved parents [7], provide a great rationale for the development of machine learning to specifically locate children with a history of parental incarceration.

The first step to validating a machine learning model for exposure to parental justice involvement is understanding whether it can accurately identify the exposure. The development of such a validated model could address research gaps and provide a foundation for exploring how data science can be leveraged to locate other at-risk groups in the pediatric electronic health record. This manuscript describes such a process and validation in hopes to inspire others to think creatively about how to address gaps in our understanding of various types of childhood adversities, specifically on the health of children of justice-involved parents and other at-risk pediatric groups. Doing so creates a novel way to apply these tools to promote child health equity.

## Methods

### Overview

In this work, we trained a state-of-the-art natural language processing model to automatically retrieve patient notes that contain evidence of parental incarceration. First, we located patient notes that are likely to contain such evidence via a keyword search. Then, we manually reviewed and labeled a large sample of those notes with respect to whether they actually identify the patient as being exposed to justice-involved parents. Finally, we used this labeled data set of notes to train and validate a model that classifies notes as true exposure to a history of parental justice involvement versus no exposure. All study procedures were reviewed and approved by Nationwide Children’s Hospital Institutional Review Board.

### Setting

We queried EPIC medical records on 1.2 million youth under 18 years of age in the electronic health record database of a large, urban, Midwestern, pediatric hospital-based institution from 2011-2019. The hospital-based system is one of the largest institutions in the United States and includes a network of 13 primary care centers, behavioral health clinics, 7 urgent care clinics, two emergency departments and 527 inpatient beds on the main campus, plus 146 offsite inpatient beds as part of its neonatal network. The institution provides care for about 1.3-1.5 million patient visits annually, including roughly 89,000 annual primary care visits. Medicaid is the primary insurer for half of all patients seen, and nearly 80% of the patients are seen within primary care. Approximately 56% of the current total pediatric population self-identified or family-identified as White, 22% identified as Black or African American, 7% identified as Latino, 3% identified as African, 4% identified as Asian, and 6% identified as Biracial/multi-racial. In addition, English-speaking patients comprised 86% of the total pediatric population, followed by Spanish (5%), Somali (3%), Nepali (1%), and “all other” languages (4%). These racial and ethnic demographic characteristics are in line with the total population characteristics of the city in which this health institution is located.

### Selection of Bidirectional Encoder Representations from Transformers

BERT (Bidirectional Encoder Representations from Transformers) is a state-of-the-art natural language processing model based on a neural network (deep learning). It is unique in its ability to pick up contextual information within and across sentences [30] The BERT model expands on the idea of context-free word embeddings (such as word2vec) by quantifying each word within its textual context using a transformer network with attention mechanism. The BERT model also utilizes self-attention to weight its input features (represented as contextualized word tokens). In practice, BERT uses a neural network to create a numerical representation of a chunk of text up to approximately 500 words long, which can then be used for classification.

### Query Details and Data Preparation

We conducted an automated search over the free-text clinical notes available within EPIC [31]. Any type of clinician note from any type of medical provider was eligible. Information about personal or familial incarceration is not routinely asked about, and providers were not mandated to document such information. We chose terms to capture the four primary types of justice involvement following arrest in the United States (eg, jail, prison, parole, and probation). Therefore, our text search first identified any note that contained at least one of the following familial terms (“mother” or “mom” or “father” or “dad” or “parent” or “grandpa” or “grandma” or “grandparent”), and at least one of the following justice terms (“prison” or “sentenced” or “incarcerated” or “probation” or “parole” or “jail”). We included grandparent familial terms as previous research via the Bureau of Justice Statistics found that nearly 45% of incarcerated mothers in state prisons and 12% of incarcerated fathers had their children cared for by grandparents during their incarceration [32]. We subsequently filtered out duplicate notes, notes that used justice terms only as part of a default screening sentence (eg, a tuberculosis risk assessment screening that clued providers about how “incarcerated adolescents” is a high-risk group), and notes in which familial terms and justice terms were more than 500 words apart (to comply within the computational requirements in order to apply BERT).

To prepare the notes for training and processing, we broke down each note into individual words (tokenization). The note was then reduced to the 500 tokens (words) window containing the maximal amount of justice keyword terms. The resulting note snippets were then used to train and evaluate the BERT model. To begin our training process, we randomly sampled 7500 notes for manual annotation. Previous work has shown the BERT model can perform well on similar tasks when fine-tuned with as few as 5600 examples [30]. We used a sample size of 7500 to allow for a similar fine-tuning sample size alongside a larger testing and validation sample size for a more robust evaluation. We compiled the notes into a secure database (REDCap Survey) and highlighted the associated familial/justice keywords. A trained undergraduate student manually reviewed and annotated each note as a true or false case of parental justice involvement. To decrease error in inaccurate annotation, the student was able to flag a note to prompt the first author (a previous prison nurse familiar with justice-based language) to review if assistance was needed in deciphering whether a note contained a true case of parental justice involvement. In addition, the first author randomly selected 500 notes (using a random number generator via Python) to verify appropriate annotation for parental justice involvement. Along with parental justice involvement, we also recorded other types of familial or personal justice-system involvement (eg, by a different family member), if applicable.

### Model Development and Training Plan

We used a publicly available BERT implementation [30] that was pretrained with a large corpus of clinical notes [33]. We adjusted the neural network output to perform a binary classification and then fine-tuned the whole network with our data set of notes that contained documented exposure to parental justice involvement. To avoid overfitting, we used 80% of the data for training, 10% for internal validation (ie, determining the number of training epochs), and 10% to test model performance. To increase robustness against the inherent randomness of neural network optimization, we repeated this process in a 10-rep, 10-fold cross-validation scheme (a common split for model training) [34] and reported average results across all folds and repetitions.

### Statistical Analysis for Algorithm Performance

We begin by reporting descriptive statistics of the manual review of the 7500 notes. In addition, we also report the average number of total words per clinician note and the percentage of notes containing each of the keywords of interest stratified by evidence of justice involvement (all notes, clinician notes with evidence of justice involvement, and clinician notes with no evidence of justice involvement). Then, we evaluate the BERT model in terms of its precision (or positive predictive value) and recall (or sensitivity). Precision is measured as the fraction of notes retrieved by the algorithm (true positives and false positives) that actually contain evidence of parental justice involvement according to our chart review (true positives). Recall is measured as the fraction of all notes with evidence of parental incarceration (true positives and false negatives) that is retrieved by the algorithm (true positives). We report the precision-recall curve (averaged across 10 training repetitions), as well as the ideal F1 score (a balanced measure of recall and precision). Finally, we use our additional manual chart review to report descriptive statistics of the false positive notes retrieved by our model.

## Results

### Keyword Search and Manual Chart Review Annotation Results

**Figure 1:**
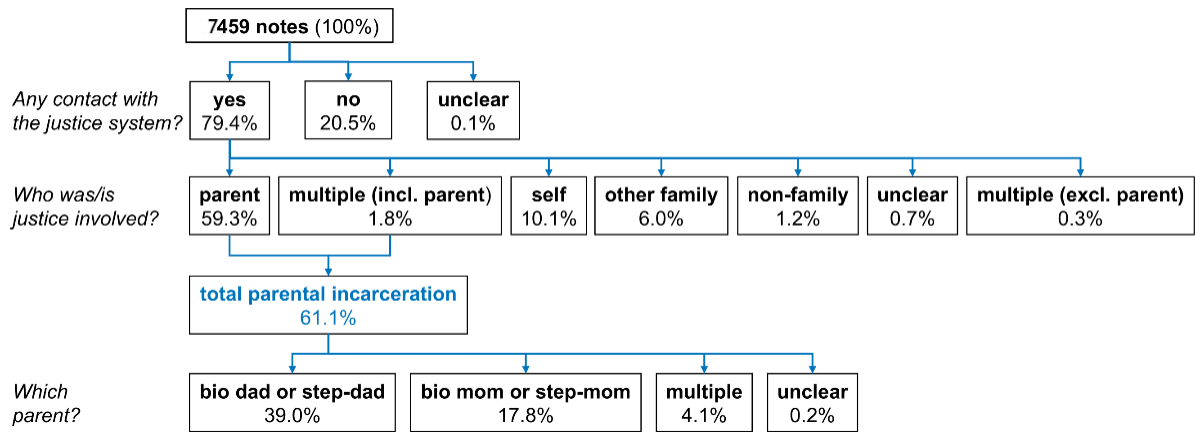
Manual chart review results from a sample of notes that matched our keyword search (any clinician note that contained at least one familial term (“mother” or “mom” or “father” or “dad” or “parent” or “grandpa” or “grandma” or “grandparent”), and at least one justice term (“prison” or “sentenced” or “incarcerated” or “probation” or “parole” or “jail”). All percentages are relative to 7459 notes.

Approximately 0.2% of the total clinician notes (N=133,211) contained the justice and family keywords and resulted in about 38,614 unique patients (or 3.30% of the total patient population during 2011-2019). Figure 1 summarizes the results of our manual review of 7459 randomly selected clinician notes (after 41 duplicate notes were excluded). Of these 7459 notes, 5926 (79.4%) notes contained evidence that the patient had exposure to some type of contact with the justice system (personal, familial, and nonfamilial). The majority (4554/7459, 61.1%) of the notes indicated exposure to a parental justice involvement (biological or step), followed by self or personal justice involvement (57/7459, 10.1%) and other family member justice involvement (451/7459, 6.0%). Paternal (biological or step) involvement with the justice system was found in approximately 2909 (39.0%) notes, while maternal (biological or step) involvement was found in 1328 (17.8%) notes, and 306 (4.1%) notes indicated more than one parent. In addition, less than 4 (0.1%) of the notes were flagged by the annotator as “unclear” and verified as “unclear” by the first author. Of the 500 notes that were randomly selected to be reviewed by the first author to assess the accuracy of notation, none were annotated incorrectly for parental justice involvement exposure. These results suggest that our initial keyword search was effective at retrieving notes of interest but also retrieved a large proportion of “false positives,” which we aimed to further filter out by training a natural language processing model.

Figure 1) Manual chart review results from a sample of notes that matched our keyword search (any clinician note that contained at least one familial term (“mother” or “mom” or “father” or “dad” or “parent” or “grandpa” or “grandma” or “grandparent”), and at least one justice term (“prison” or “sentenced” or “incarcerated” or “probation” or “parole” or “jail”). All percentages are relative to 7,459 notes. Table 1 outlines the clinician note characteristics such as the average number of total words per note and the percentage of notes containing each of the keywords of interest stratified by evidence of any type of justice involvement. The average number of total words per clinician note that contained evidence of justice involvement was higher than the average word count per note of those that did not contain evidence of justice involvement (1121.9 words per note compared to 977.6 words per note, respectively). In addition, notes with evidence contained a higher percentage of all of the family-related keywords, with “mother” being the most frequent family term. The family terms “grandpa,” “grandma,” and “grandparent” were twice as frequent in the notes that contained evidence of justice involvement. In addition, the most frequent justice-related keyword was “incarcerated.” The justice-related keywords “jail” and “sentenced” appeared more frequently in notes that contained no evidence.

**Table 1:** Clinician note characteristics, including the average number of words and percentage of keywords in each clinician note.

Clinician note characteristics		Total clinician notes (N=7459)	Clinician notes with evidence of any type of justice involvement (n=5926)	Clinician notes with no evidence of justice involvement (n=1529)
Number of words per note, mean (SD)		1084.3 (776.5)	1121.9 (781.3)	977.6 (752.5)
**Notes containing family keywords (%)**				
	Mother	86.3	88.4	78.0
	Father	67.3	71.9	49.6
	Parent	62.6	63.7	58.1
	Mom	56.3	57.2	53.0
	Dad	36.6	38.5	29.2
	Grandpa	9.1	10.3	4.7
	Grandma	7.6	8.4	4.6
	Grandparent	7.2	8.1	3.7
**Notes containing justice keywords (%)**				
	Incarcerated	41.0	43.3	32.2
	Jail	37.1	34.6	46.8
	Prison	20.7	22.4	14.2
	Probation	15.9	18.2	7.0
	Parole	2.2	2.5	1.0
	Sentenced	1.6	1.4	2.5

### Model Performance

**Figure 2:**
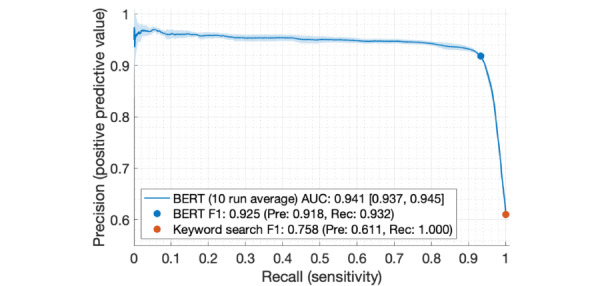
Cross-validated precision-recall curve for identifying notes with evidence of parental justice involvement for the BERT model, compared to keyword search. The curve depicts average performance of 10 independent training runs, with shaded areas indicating a 95% CI. AUC: area under the curve; BERT: Bidirectional Encoder Representations from Transformers; Pre: precision; Rec: recall.

Figure 2 summarizes the note retrieval performance of our keyword search and subsequent BERT model. Under the assumption that the keyword search is perfectly sensitive (ie, it is unlikely that a note contains clear evidence of parental justice involvement but at the same time does not contain any of our keywords), the keyword search alone can be considered as having a recall of 1, precision of 0.611, and a resulting F1 score of 0.758. Application of the BERT model on all retrieved notes increased overall precision while only sacrificing a small amount of recall. For example, a decision threshold that optimizes BERT's F1 score to 0.925 results in a precision of 0.918 (50.2% increase) and a recall of 0.932 (6.8% decrease).

### False Positive Analysis

Utilizing the note annotations summarized in Figure 1, we found that 53.8% (208/387) of all notes that our BERT model falsely flagged for exposure to parental justice involvement still contained evidence of other types of contact with the justice system (eg, sibling, self, etc). This percentage was higher than the baseline proportion of such notes that were retrieved by the keyword search (1324/7459, 17.6%).

## Discussion

### Principal Findings

In this paper, we applied the use of natural language processing (NLP) and machine learning to locate children ever exposed to parental justice involvement in the electronic health record of a large Midwestern pediatric health system—an innovative approach to aggregating health data on an understudied and stigmatizing childhood adversity. The use of machine learning greatly improved the precision of locating children who have justice-involved parents from 61% (using a keyword search) to 92%. To our knowledge, only one study has validated the use of NLP to locate adults with a history of personal incarceration using the Veterans Administration health record.[28] In their study, the NLP keyword search resulted in an F1-score (a balanced measure of recall and precision) of 0.58; and after integrating NLP and a simplistic machine learning approach, the F-1 score improved to 0.75 [28]. Our study achieved a similar increase, but our keyword search resulted in an F-1 score of 0.76, and after integrating BERT, the F-1 score improved to 0.93.

Our findings also revealed that when notes were falsely flagged by the model for exposure to parental justice involvement, a much higher percentage of notes flagged still contained evidence of another type of contact with the justice system compared to such notes located by the basic keyword search. In addition, compared to notes with no evidence, clinician notes with evidence of justice involvement were slightly longer and had a higher frequency of all family- and justice-related keywords except for the justice keywords “jail” and “sentenced.” This may relate to the number of notes that contained evidence of personal youth involvement with the justice system, rather than parental (as youth typically have shorter sentences that align with “jail”). Importantly, the grandparent-related terms were nearly double in frequency and are in line with research noting that nearly half of all youth with incarcerated mothers are cared for by grandparents [32]. Other keywords such as “caregiver” and “legal guardian,” “justice,” “legal,” and “crime/criminal” may also be important to include in future research.

We are among the first to leverage data science approaches to address gaps in the pediatric health sciences related to underrepresented groups of youth. The total time for the development of our machine learning model included several weeks to annotate clinician notes, 2 months of data scientist work, and cost about $12,000 at this particular institution. The computer code associated with this model will remain publicly available at no cost to those who are interested in testing its application in other pediatric electronic health record systems [35] (please email if the web address embedded in the citation becomes faulty). Cost-effective, less-invasive, and time-saving approaches to cohort identification could surely advance our understanding and advocacy of historically marginalized and underrepresented groups of youth and families. The child health consequences of complex social phenomena such as mass incarceration must be explored, and efficient approaches to recognition in the clinical setting can aid in that process as we await wide-scale screening of childhood adversities within health care systems and settings.

Further validation with multiple data sources is needed (eg, comparison of findings to those youth identified with exposure to parental justice involvement using adverse childhood experiences screening tool checklists, or other cross-sector administration data to verify parental contact with the system) to strengthen its future use and will be an important next step. Eventual integration of these models in larger pediatric learning health systems such as PEDSnet (a multi-specialty network that conducts observational research and clinical trials across multiple children's hospital health systems) [36] could also be explored to understand whether differences in care and health care use exist for youth who have a justice-involved parent across systems. Once these models are extensively researched and validated, the use of these techniques could extend beyond cohort identification and eventually be used to link families to supportive behavioral health treatment, case management social services, and other positive prosocial or community resources to mitigate child stress and adversity.

The underlying approach has the potential to be extended to the identification of other types of childhood adversity (eg, sex trafficking). Until routine screening for adverse childhood experiences becomes commonplace, artificial intelligence could be an important tool to accelerate efforts for greater understanding of at-risk populations. Implications for doing so are great, as better science and greater understanding of children of justice-involved parents could spur greater investment and intervention development designed to improve their health and well-being and decrease their risk for future justice system involvement. As NASEM recommends in their latest report on increasing opportunity for all youth, we need “greater collaboration among our health, justice, and child welfare systems to transform child health”[11]. We feel strongly that the use of artificial intelligence within pediatric health settings could accelerate these collaborative cross-sector efforts.

It is important to note that the use and application of artificial intelligence and algorithms to address the needs of our justice system (eg, risk prediction and prediction of public threats to safety) are widely investigated and contested [37]. The use of such algorithms in pediatric health care settings to identify and locate patients with varying exposures to the justice system is novel and certainly warrants similar investigation and ethical scrutiny. It would be important to consider when, why, and who should be able to access and use data on health-related social risk factors such as familial justice involvement [38]. While all personal arrests and incarcerations are public knowledge, the use of this information in pediatric research is novel. Most institutional review boards have additional regulatory procedures or special review processes to ensure protections of justice-involved youth and adults in research, but youth who have family members who are justice-involved are not typically considered. Addressing the ethical challenges related to the development and implementation of machine learning to identify children of justice-involved parents is imperative and necessitates the engagement and involvement of these youth and their caregivers. Future research in this area could benefit from comparative investigations of other types of machine learning models and the integration of emerging frameworks designed to facilitate responsible and ethical digital technology for research purposes [39,40]. More research is also needed (and underway) to better understand family perceptions and attitudes surrounding the use of artificial intelligence to mine sensitive and stigmatizing information even amid the best intentions of bettering care and assisting these youths.

### Limitations

Our study is not without limitations. It is important to note that all estimates of youth exposed to parental justice involvement are unverified and only captured (1) families that disclosed and (2) a provider who was willing to document the exposure. Potential selection biases in our model may exist as there may be differences in providers who ask about justice involvement compared to those who do not, providers who choose to record the information received compared to those who do not, and families who feel comfortable or safe providing such information compared to those who feel discriminated, ostracized, ashamed, or stigmatized in systems not designed to support or assist families affected by justice involvement. Our results likely underestimate the total number of children who have experienced justice-involved parents seen in our system and may represent a subset for which clinicians have a higher index of suspicion. Even given the potential biases in the identified population, our model improves the accuracy of locating patients who are apt to disclose parental justice involvement (with or without direct questioning of a provider) and allows identification of a high-risk cohort for research. Last, we were unable to verify our machine learning algorithm with the “ground truth” because screening for adverse childhood experiences such as parental incarceration is not routinely conducted in any setting of care within this institution. Apart from these important limitations, we feel our study has provided an important innovation to pediatric research.

### Conclusions

Machine learning is a novel cohort identification method that may be able to fulfill the gaps in the sciences related to our understanding of the health of children of justice-involved parents. Doing so could inform intervention development and effective policy creation to improve the cross-sector care and health of children of justice-involved parents—and other youth with various types of justice system involvement.

## Abbreviations

BERT: Bidirectional Encoder Representations from Transformers
NASEM: National Academies of Sciences, Engineering, and Medicine
NLP: natural language processing
